# Supplementary material for: Differential transcript profile of inhibitors with potential anti-venom role in the liver of juvenile and adult Bothrops jararaca snake
Source: PeerJ. 2017 Apr 27;5:e3203. doi: 10.7717/peerj.3203 (PMC5410159; doi:10.7717/peerj.3203)
Supplement: Data S2 [file peerj-05-3203-s002.doc]

**Supplementary data S2**. Primers used for qPCR.

| **Gene** | **Primer** |
| --- | --- |
| β- actin *foward* | 5’-GGCCAACAGAGAGAAGATGACCC-3 |
| β- actin *reverse* | 5’-TCGGTCAAGTCACGGCCA-3’ |
| Bj46a *foward* | 5'-TCAAGAGGGCAGCACAAGAAT-3' |
| Bj46a *reverse* | 5'-AGTCCGACTCAAACTGTTCATC -3' |
| PLI-γ *foward* | 5'-CCAGAAGATGTATGTGGCAAGG -3 |
| PLI-γ *reverse* | 5'-TTTGGTCGGGAGAGGGGC -3' |
| C1- foward | 5’-TCGCTCCAATGAACCAGTCG-3’ |
| C1-reverse | 5’-TGACCCGTCCCAGAAAGATTG-3’ |
| Inter-alpha inhibitor foward | 5’- CTTACCTCACCATTCAACAACTTCT-3’ |
| Inter-alpha inhibitor reverse | 5’- TGGACCCTTGCTGCTTTGC-3’ |
