# Supplementary material for: Differential transcript profile of inhibitors with potential anti-venom role in the liver of juvenile and adult Bothrops jararaca snake
Source: PeerJ. 2017 Apr 27;5:e3203. doi: 10.7717/peerj.3203 (PMC5410159; doi:10.7717/peerj.3203)
Supplement: Data S3 — Multiple alignment of amino acid sequences of Bj429, Bj46a from Bothrops jararaca (AAG09055.1), HSF (BAB39858.1) and HSF-like (BAD88535.1) from Protobothrops flavoviridis and MSF (Q5KQS4.1) and HLP-B (Q5KQS2.1) from Gloydius brevicaudus. Identical residues among Bj429 and anti-hemorrhagic molecules (MSF, BJ46a and HSF) are highlighted in blue; identical residues among Bj429 and molecules with no anti-hemorrhagic activity (HLP-B and HSF-like) are highlighted in yellow; and residues exclusive of Bj429 are highlighted in green. Gray shadow indicates Bj429 residues described in molecules with and without anti-hemorrhagic activity (but not in all of them). [file peerj-05-3203-s003.docx]

Bj429 ------------------------------------------------------------

HLP-B MNSLVALVLLGQMIGSTLSHHLQSHVDCNGEDAEKWADMAVHYINEHNLHGYKQVFNVIN

HSF-like MNSLVALVLLGQIIGSTVSFQLGPNMDCNTKGTKDWADIGVHYINEHKLHGYKQALNVIK

MSF MHFLVALVLLGQIIGSTLSSQVRGDLECNDREAKEWADQAVRYINEHKLHEYKQALNVIK

Bj46a MNSLVALVLLGQIIGSTLSSQVRGDLECDEKDAKEWTDTGVRYINEHKLHGYKYALNVIK

HSF MNSLVALVLLGQIIGSTLSSQVRGDLECDDKEAKNWADDAVRYINEHKLHGHKQALNVIK

Bj429 ------------------------------------------------------------

HLP-B EIHVLPRRPRGKIIILELKLLETECHVLDPTPVENCTVRPPHYHAVEGDCDVKILHDEG-

HSF-like IFRLLPSDGRSVIFHFNLNLLETECHVLDSTPVENCTVRPQHNHAVEMDCNVRIIHDITT

MSF NIVVVPWNGDLVAVFLKLNLLETECHVLDPTPVEKCTIRPQQNHAVEMDCDAKIMFDVET

Bj46a NIVVVPWDGDWVAVFLKLNLLETECHVLDPTPVKNCTVRPQHNHAVEMDCDVKIMFNVDT

HSF NICVVPWNGDLVAVFLELNLLETECHVLDPTPVEKCTVRQQHNHAVEMDCDAKIMFNVET

Bj429 --------------GVEDVRRNCPKCPLLLNLNDPQVVDSVEYVLNKHNEKVSGHVYEVL

HLP-B VDKVIGAKCHSDPDSVEDVRRNCPKCPILLPLSDPHVVDSVEYVLNKHNEKLSGHVYEVL

HSF-like FEDEVFVKCSSTPGSVENILRDCPKCPILLSPNDPHVVDSVEYVLNKHNEKLSGHIYEVL

MSF FKQDVFVKCHSTPDSVEDVRRNCLKCPILLSPSDPHVVDSVEYVLNKHNEQLSGHVYEVL

Bj46a FKEDVFAKCHSTPDSVENVRRNCPKCPILLPSNNPQVVDSVEYVLNKHNEKLSDHVYEVL

HSF FKRDVFVKCHSTPDSVENVRRNCSKCPILLPPNNPHVVDSVEYVLNKHNEKLSGHIYEVL

.**:: *:* ***:** .:*:**************::*.*:****

Bj429 EISRGQHKNEPEAYYVEFAIVEVNCTAQEAHDDHHQCHPNTAGENHIGFCRATVFRSHAS

HLP-B EISRGQHKYEPEAFYVEFAIVEVNCTAQEAHDDHHHCHPNTAGENHIGFCRATVFRSHAS

HSF-like EISRGQHKYEPEAYYLEFVIVEINCTAQEAHDDYHQCHPYTAGEDHIAFCRSTVFRSHAS

MSF EISRGQHKYEPEAFYVEFAIVEVNCTAQEAHDDHHHCHPNTAGEDHIAFCKATVFRSHAS

Bj46a EISRGQHKYEPEAYYVEFAIVEVNCTAQELHDDHHHCHPNTAGEDHIGFCRATVFRSHAS

HSF EISRGQHKYEPEAYYLEFVIVEINCTAQEAHDDHHQCHPYTAGEDHIAFCRSTVFRSHAS

******** ****:*:**.***:****** ***:*:*** ****:**.**::********

Bj429 LEKPKDEQFESDCVIFDVKDGHAHSHLIEHHVGKYSTSPGHNNTVLNLVHSHNHTSASHE

HLP-B LEKPKDEQFESDCVIFDVKEGHAHSHLIEHHIGNYNTSPGHNNTVLNLAHSHNHTSASHE

HSF-like LEKPKDEKFESDCVILDVKEGHAHSHLIEHHVGKYSTSPGYNST----------------

MSF LEKPKHENFESDCVILDVKEGHAHSHLIEHHIGKYSTSPGQNST----------------

Bj46a LEKPKDEQFESDCVILHVKEGHAHSHLIQQHVEKDSISPEHNNTALNFVHPHNDTSTSHE

HSF LEKPKDEKFESNCVILDVKDGHAHSHLIQQHIEKNSISPEHNITILNFVHPDDHTSTSHE

*****.*:***:***:.**:********::*: : . ** * *

Bj429 SHSHEHVTEVPVAVAKREVPKDVPHDHTHPVKLCPGKVHHFEL-

HLP-B SHSHEHVAEVPVAVAKREVPTNTPHDHTHPVKLCPGKVHHFKL-

HSF-like ---DECVVECPVAFVNKEVPTDISDHNTPPVKGCPGRVLHFQL-

MSF ---VECVAECPVAFVNKEVPTDISDRHTTPVKGCPGKILHFQL-

Bj46a S--HEHLAEVPVAFVKKELPKDISDRHTTPVKGCPGKVHHFEL-

HSF S--HEHVAEVPVVFVKKELPTDISDHHTTPVKGCPGKVHHFKLY

* :.* **...::*:*.: . :* *** ***:: **:*

**Supplementary data S3.** **Multiple alignments of amino acid sequences of Bj429 with similar sequences described in different species of snakes.** Multiple alignment of amino acid sequences of Bj429, Bj46a from *Bothrops jararaca* (AAG09055.1), HSF (BAB39858.1) and HSF-like (BAD88535.1) from *Protobothrops flavoviridis* and MSF ([Q5KQS4.1](https://www.ncbi.nlm.nih.gov/protein/82126270?report=genbank&log$=prottop&blast_rank=1&RID=96T2H6FZ014)) and HLP-B (Q5KQS2.1) from *Gloydius brevicaudus*. Identical residues among Bj429 and anti-hemorrhagic molecules (MSF, BJ46a and HSF) are highlighted in blue; identical residues among Bj429 and molecules with no anti-hemorrhagic activity (HLP-B and HSF-like) are highlighted in yellow; and residues exclusive of Bj429 are highlighted in green. Gray shadow indicates Bj429 residues described in molecules with and without anti-hemorrhagic activity (but not in all of them).
